# Synthesis of Functionalized Carboxylated Graphene Oxide for the Remediation of Pb and Cr Contaminated Water

**DOI:** 10.3390/ijerph191710610

**Published:** 2022-08-25

**Authors:** Sana Farooq, Humera Aziz, Shafaqat Ali, Ghulam Murtaza, Muhammad Rizwan, Muhammad Hamzah Saleem, Shahid Mahboob, Khalid A. Al-Ghanim, Mian N. Riaz, Behzad Murtaza

**Affiliations:** 1 Department of Environmental Sciences and Engineering, Government College University, Faisalabad 38040, Pakistan; 2 Department of Biological Sciences and Technology, China Medical University, Taichung 40402, Taiwan; 3 Institute of Soil and Environmental Sciences, University of Agriculture, Faisalabad 38000, Pakistan; 4 College of Plant Science and Technology, Huazhong Agricultural University, Wuhan 430070, China; 5 Department of Zoology, College of Science, King Saud University, Riyadh 11451, Saudi Arabia; 6 2476 TAMU, Texas A&M University, College Station, TX 778, USA; 7 Department of Environmental Sciences, Vehari-Campus, COMSATS University Islamabad, Vehari 61100, Pakistan

**Keywords:** carboxylated graphene oxide, heavy metals, water, remediation, adsorption kinetics

## Abstract

With the growing scarcity of water, the remediation of water polluted with heavy metals is the need of hour. The present research work is aimed to address this problem by adsorbing heavy metals ions (Pb (II) and Cr (VI)) on modified graphene oxide having an excess of carboxylic acid groups. For this, graphene oxide (GO) was modified with chloroacetic acid to produce carboxylated graphene oxide (GO-COOH). The successful synthesis of graphene oxide and its modification has been confirmed using Fourier transform infrared spectroscopy (FT-IR), Raman spectroscopy, X-ray Diffraction (XRD), Scanning electron microscopy (SEM), Energy Dispersive X-ray Analysis (EDX) and Transmission electron microscopy (TEM). The increase in surface area of graphene oxide after treatment with chloroacetic acid characterized by BET indicated its successful modification. A batch experiment was conducted to optimize the different factors affecting adsorption of both heavy metals on GO-COOH. After functionalization, we achieved maximum adsorption capacities of 588.23 mg g^−1^ and 370.37 mg g^−1^ for Pb and Cr, respectively, by GO-COOH which were high compared to the previously reported adsorbents of this kind. The Langmuir model (R^2^ = 0.998) and Pseudo-second-order kinetic model (R^2^ = 0.999) confirmed the monolayer adsorption of Pb and Cr on GO-COOH and the chemisorption as the dominant process governing adsorption mechanism. The present work shows that the carboxylation of GO can enhance its adsorption capacity efficiently and may be applicable for the treatment of wastewater.

## 1. Introduction

Due to the scarcity of freshwater availability, the utilization of municipal wastewater in agricultural land has now become a conventional practice. Accumulation of heavy metals by released untreated industrial effluent to watercourses is a major environmental issue in the present era [1]. Human society is facing several hygienic, health and environmental issues due to the toxicity of these metal ions, as these are non-biodegradable, having the potential to bioaccumulate and may prove carcinogenic [2].

The wastewater from several industries, such as fertilizers, tanneries, paper, sugar, mining, alloying, electroplating, batteries, metal fabrication, pigment, petrochemicals, and paints is a source of various toxic metals. Certain heavy metals such as lead (Pb) and chromium (Cr) present in even in trace amounts, are very toxic. Pb (II) is responsible for several health issues in kids with their age under six years, such as learning disabilities, less IQ, behavioral problems, attention deficit disorders, impaired hearing, and retarded growth [3]. Chromium is carcinogenic and can also cause skin, respiratory issues, mutagenicity, and neurological deformities [4].

Previously, various physical and chemical processes have been proposed for the remediation of heavy metal polluted water, such as solvent extraction, chemical precipitation, membrane separation and ion-exchange [5]. Adsorption is most widely employed because of its ease, elasticity, sensitivity towards toxic substances, and high efficacy for applications on large scale. It is cost effective and economically viable alternative for extracting metals from water. Adsorbate is a substance that adheres to solid surfaces and solid surface called adsorbent. The contaminants in the solution are adsorbed over the active-sites available on the surface of adsorbent [6,7]. The adsorption capacity of solid adsorbents might be increased either by improving the surface area via providing more adsorption sites [8,9,10] or more effectively by modifying the surface properties of solid adsorbents by introducing new functional groups such as amino, carboxyl etc., on their surfaces, which can significantly enhance the adsorption capacity by complexing with heavy metals [11].

Graphene-oxide (GO), a new carbon-based adsorbent has gained considerable attention as an adsorbent for heavy metals, dyes and ions owing to its efficient dispersion formation in water, cost effectiveness, easy preparation methods and biocompatibility. GO has distinctive physicochemical properties such as high specific surface area, greater adsorption power and incredible mechanical strength [12]. Graphene oxide and its derivatives are being used in environmental fields to make novel adsorbents for contaminant monitoring or removal [12] for example removing fluoride ions [13] from the contaminated water as well as different heavy metals such as Pb, Cd and As [14,15]. The basal plane of GO has oxygen containing hydroxyl (-OH) and epoxy groups, whereas on the edges, some carboxylic functional groups are also present. These functional groups enable GO to bind to several inorganic and organic species by hydrophobic interactions, π-π stacking and hydrogen bonding [16].

Functionalization of GO has emerged as a new class of materials. There are mainly three methods described in the literature for the functionalization of GO i.e., covalent functionalization, non-covalent functionalization, and element doping. Significant research has been conducted to produce GO derivatives with different functional groups such as amine, sulfur, halogens, carboxylic, mercapto, and many others. The family of functionalized GO has grown significantly during the last few years. Functionalized GO is fascinating because it provides a wide range of possibilities to modify the chemical and physical characteristics of GO by attaching a specific kind and quantity of organic or inorganic moieties to either the graphene basal plane or its edge [17]. Researchers functionalized GO with amine [18], sulfur [19], carboxylated [20], halogens [21], mercapto [22], and phosphate groups [23] and obtained outstanding results for different applications.

Functionalization of GO with different groups such as aminated [24], mercapto [25], carboxylated [26], and sulfur [27] has been widely used for the adsorption of different heavy metals. Fang et al. modified GO with amine groups to adsorb the cobalt ion and their results reveal that aminated GO could be removed more than 90% of Co (II) ions in just 5 min for diluted solutions [24]. Pirveysian and Ghiaci used sulfur functionalized GO for the sorption of different heavy metals (Zn^2+^, Ni^2+^, Cd^2+^ and Pb^2+^) and their results proved that sulfur GO can effectively adsorb heavy metals [25]. Mercapto functionalized GO was also has been implied for the wastewater treatment and their findings proved the mercapto functionalized GO is an efficient adsorbent for mercury [27]. However, carboxylation of GO is highly efficient for the adsorption of heavy metal ions because carboxylic group consists of two main functional groups namely carbonyl and hydroxyl which are bounded by the same carbon atom. This distinctive feature between two groups forms polar weak acid group. Furthermore, during deprotonation, the resonance over the two O-atoms stabilized the carboxylate (COO-) anion enabling the carboxyl group to adsorb more pollutants by making an efficient complex [26].

Chloroacetic acid has been effectively implied for the conversion of hydroxyl groups of GO into carboxylic groups. In this way after carboxylation, the surface of GO is enriched with carboxylic groups, and it has been widely used for various applications such as photocatalytic activity [28], and metal adsorption e.g., Cu, Pb, Hg and U [29,30,31,32].

The present study was designed to study the effect of carboxylic acid groups on the surface of GO. For this, GO has been synthesized using modified Hummer’s process which has been confirmed by Raman Spectroscopy and XRD. The synthesized GO was further modified to generate carboxylic acid groups to enhance its capacity to remove heavy metals such as Pb and Cr which are causing toxicity to drinking water worldwide.

## 2. Materials and Methods

### 2.1. Chemicals

Graphite powder (99.99%), H_2_SO_4_ (98%), sodium nitrate (98%), hydrogen peroxide (30%), potassium permanganate (99.7%) and NaOH (98%) were procured from Sigma-Aldrich. Chloroacetic acid (99%) was procured from Tokyo Chemical Industry Co., Ltd. A standard stock solution (1000 mg L^−1^) 19.61 mM L^−1^ of chromium (Cr) and 4.83 mM L^−1^ lead (Pb) was prepared by dissolving Cr(NO_3_)_3_ 9H_2_O (98%) or Pb(NO_3_)_2_ (98%) in deionized water.

### 2.2. Synthesis of Graphene Oxide (GO)

Graphene oxide was synthesized by oxidizing the graphite powder according to Hummer’s method [33,34]. Firstly, 5 g of graphite powder was taken in 500 mL flask followed by the addition of 2.5 g sodium nitrate and 250 mL sulfuric acid. Thereafter, 30 g of KMnO_4_ was slowly added under constant mechanical stirring and kept the temperature of the reaction below 10 °C. After the addition of KMnO_4_, the color of the mixture turned green, and the temperature was raised to 35–40 °C until the completion of oxidation process. Afterward, the color of mixture changed into light brown which confirms the successful oxidation of graphite. 200 mL distilled water was added to dilute the solution, and then solution was heated for 30 min to a temperature of 98 °C. To complete or stop the oxidation process of graphite, 30 mL of H_2_O_2_ was added to the cooled solution and graphitic oxide of bright yellow color was obtained. When the reaction was completed, centrifugation of graphitic oxide was conducted for 15 min, and the excess acid was removed by neutralizing the pH with distilled water. The distinct GO layers (2.7 g) were separated via sonication for 20 min, and drying was conducted at 60 °C in an oven for 24 h.

### 2.3. Modification of GO

The graphene oxide was modified by the introduction of more content of carboxyl groups. GO (100 mg) was added to the distilled water (500 mL) and the dispersion of GO was obtained by bath sonication at room temperature for 2 h. Then, GO solution was added with 10 g NaOH and 10 g chloroacetic acid (Cl-CH_2_COOH) and was sonicated for 2 h for the conversion of hydroxyl groups -OH to carboxylic groups -COOH via acetic acid moieties conjugation [35,36]. The resulting solution of GO-COOH was then neutralized with HCl and rinsed with distilled water to remove the impurities. Afterward, drying was conducted at 60 °C in an oven for 24 h and black-brown powder (50 mg) with more carboxylated content was obtained.

### 2.4. Adsorption Process

**Figure 1:**
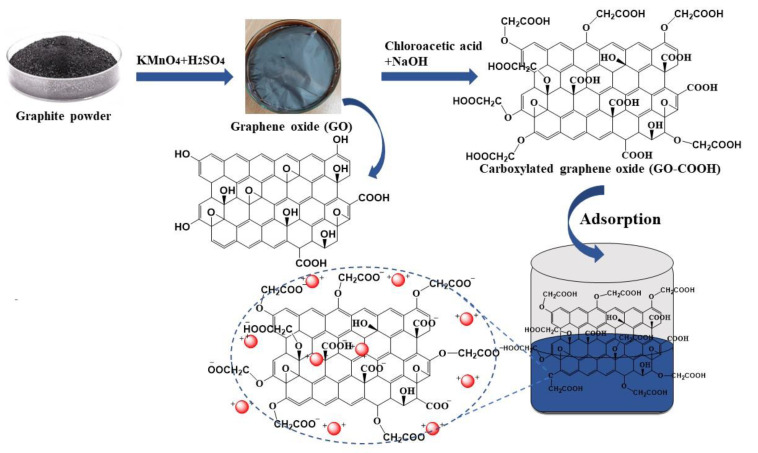
Schematic representation of synthesis of GO, its modification with chloroacetic acid and adsorption of Pb and Cr.

An adsorption experiment in batches was adopted to investigate the mechanism and adsorption capabilities of GO-COOH adsorbent on heavy metals. Pb (NO_3_)_2_ or Cr (NO_3_)_3_ were used to make stock solutions with starting concentrations ranging from 0 to 5, 25, 50, 100, 200, and 300 mg L^−1^. For such procedures, specific amount of both Pb and Cr solution were poured inside a 50 mL beaker and specified dose of adsorbent GO-COOH was added into centrifuge tubes. In order to adjust the pH, NaOH and HNO_3_ solution (0.1 mol L^−1^) were gradually added after adsorbent addition (Schematic of synthesis and adsorption is shown in Figure 1). Centrifuge tubes were shaken for 4 h in a shaker at 25 °C at the shaking speed of 180 rpm and then centrifuged for 10 min at 10,000 rpm. The adsorbed material was then removed from the solution using filter paper. The atomic absorption spectrophotometer (Thermo Electron AA, Waltham, Solar-Series MA, USA) was used to quantify Pb and Cr in the supernatant solution. All samples were run as triplicates and results were taken as average of 3 replicates. The percentage removal was determined using Equation (1) while, Equation (2) was employed to calculate the experimental adsorption capacities [37].

(1) $$\mathrm{Removal}\;(\%)=\left(\frac{C_{o}-C_{e}}{C_{o}}\right)\times 100$$

(2) $$\;Q_{e}=\left(C_{o}-C_{e}\right)\frac{V}{m}$$

where in above equations, *Q_e_* is the experimental adsorption capacity in mg g^−1^. *C_o_* (mg L^−1^) is the concentrations of Pb and Cr before the adsorption while *Ce* (mgL^−1^) represents the Pb and Cr concentrations after adsorption m shows the adsorbent weight in g while *V* expressed the suspension volume in L.

### 2.5. Analytical Methods

The presence of functional groups on GO and GO-COOH was characterized using FT-IR spectrophotometers (Perkin Elmer, Shelton, CT, USA). Raman spectra were recorded between 0 and 3500 cm^−1^ Via Raman Microscope by RENISHAW U.K. with excitation laser 514 m, laser Exposure time/s 10 s, grating 1800 I/mm objective X5 and 100% laser power. X-ray diffraction (XRD) analyses were performed on PANalytical diffractometer, using Cu Ka radiation, under a voltage of 40 kV and a current of 200 mA. The Brunauer-Emmett-Teller (BET) specific surface areas of both GO and GO-COOH was analyzed using ASAP 2460 instrument with 77 k via nitrogen adsorption desorption isotherm using a method [38]. The morphologies were characterized by HITACHI S-3500N scanning electron microscope. To further confirm the formation of sheets and to understand the difference between GO and GO-COOH, transmission electron microscopic analysis was conducted by transmission electron microscope (FEI Tecnai G2 20, Hillsboro, USA). The heavy metals Pb and Cr in the supernatant solution were identified by the atomic absorption spectrophotometer (AAS) (Thermo Electron, AA, Waltham, Solar-Series MA, USA), using graphite furnace atomic absorption spectrometry (GFAAS).

## 3. Results and Discussion

### 3.1. Characterization of GO and GO-COOH

The GO and GO-COOH were characterized for FT-IR to identify the functional groups of their surfaces. The SEM, TEM images, Raman spectroscopic analysis, and XRD were used to check surface morphology and structural changes before and after modification of GO.

#### 3.1.1. Spectroscopic Analysis

**Figure 2:**
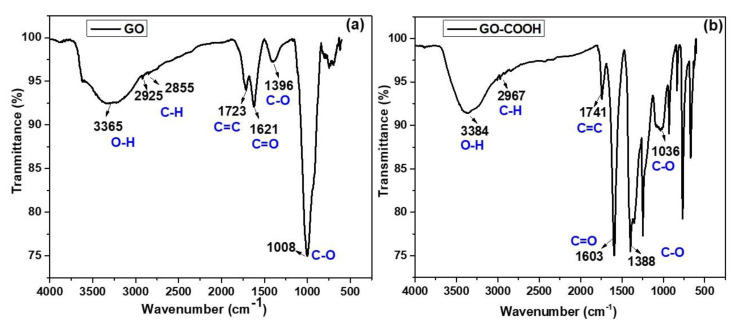
FTIR spectrum of (**a**) GO and (**b**) GO-COOH.

Figure 2 depicted the FT-IR results of GO and GO-COOH to evaluate the presence of different functional groups. FT-IR spectra of GO exhibit strong band at 3365 cm^−1^ which ascribed to the presence of the stretching mode of hydroxyl groups. The peaks at 2925 and 2855 cm^−1^ are assigned to the asymmetric and symmetric stretching mode of the C-H bond in GO structure (Figure 2a). The peak at 1621 cm^−1^ represents the carbonyl group (C=O) while the peak at 1723 cm^−1^ corresponds to the C=C of SP^2^ hybridization. The peak at 1396 cm^−1^ was assigned to the C-OH bond and the peak at 1008 cm^−1^ denotes the stretching vibrational mode of C-O bond in GO structure and our results are in accordance with the previous literature [39,40].

Although the structure of carboxylated GO is identical to the GO, with more content of carboxyl groups. Therefore, all functional groups in GO structure are also observed in the FT-IR spectra of GO-COOH (Figure 2b) but the characteristic peaks at 1603 and 1741 cm^−1^ are more intense as a result of more COOH bonds in GO-COOH structure which indicates the successful formation of carboxylic GO with chloroacetic acid, and our findings are related to the previous studies [41,42].

**Figure 3:**
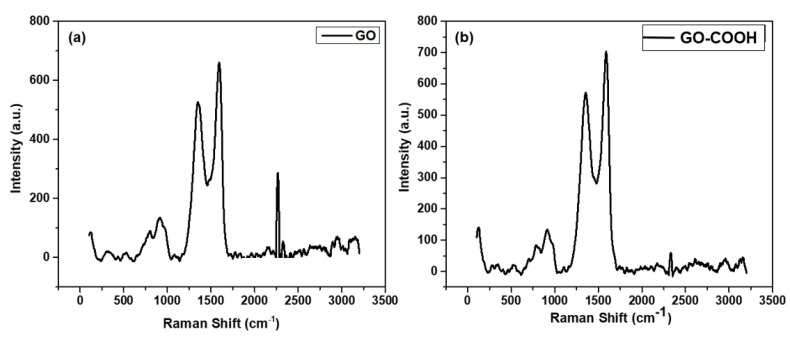
(**a**) Raman spectra of GO, (**b**) Raman spectra of GO-COOH.

The Raman spectrum of GO and carboxylated GO have been depicted in Figure 3a,b, respectively, in which two characteristic peaks of the G and D bands are present around 1580 cm^−1^ (for GO) and 1357 cm^−1^ (for GO-COOH). The vibrations of sp^2^ bonded carbon atoms in GO structure are confirmed from G band while the vibration of disordered, sp^2^-bonded carbon atoms is associated with D band. In addition, we achieved an identical ratio between the intensities of GO and GO-COOH representing bands, i.e., G and D bands indicating that the two molecules share a comparable structure, and the modified GO did not alter the structure of GO [43]. However, the Raman analysis confirms the successful modification of GO as the intensity of D band representing modified carboxylated GO is higher than the original GO [44,45].

**Figure 4:**
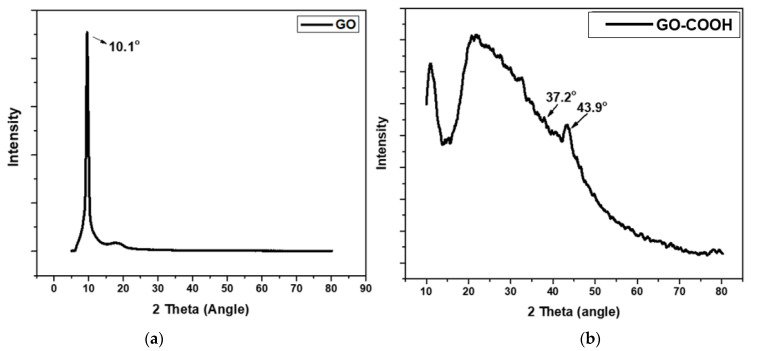
XRD analysis of GO (**a**) and GO-COOH (**b**).

The XRD analysis was performed to examine the alteration in the crystalline structure of GO before and after its modification Figure 4. The XRD spectrum of GO showed a characteristic peak at 10.1° denoting a 0.858 nm interlayer spacing of the sheets of GO. On the other hand, two additional peaks were observed in the XRD spectrum of carboxylated GO representing interplanar distance of 0.201 nm and 0.232 nm at 43.9° and 37.2°, respectively, [40] that confirms the successful formation of GO-COOH.

The surface area characterization of GO and GO-COOH showed that it has significantly increased after modification of graphene with chloroacetic acid (Supplementary Table S2). The layers of GO are always attached to each other. On modification with chloroacetic acid, the carboxylic acid group ionized to form negative ions on the surface of GO causing electrostatic repulsion among its layers and surface area is increased [29].

#### 3.1.2. Microscopic and EDX Analysis

**Figure 5:**
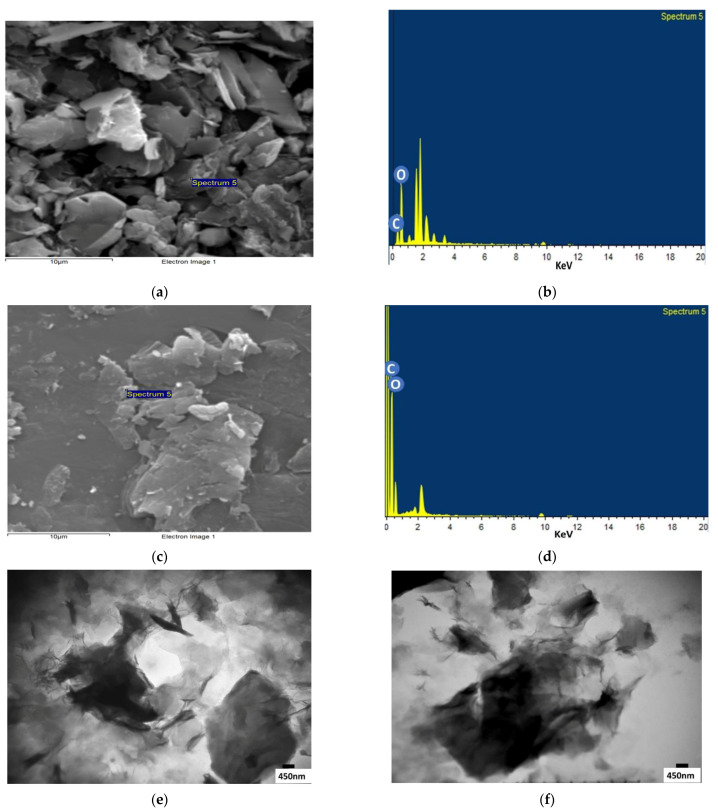
(**a**) SEM image of GO; (**b**) EDX of GO; (**c**) SEM image of GO-COOH; (**d**) EDX of GO-COOH (**e**) TEM micrograph of GO; (**f**) TEM micrograph of GO-COOH.

Morphological features of GO and GO-COOH were determined by SEM (Figure 5a,c). It can be seen clearly in Figure 3a that the structure of GO happens to be two-dimensional, similar to sheets. GO also has a plane surface, crumpled edge, and large thickness. The thickness of GO sheets increases owing to the addition of functional groups containing oxygen. It is observed that GO sheets have a bent shape, thin layers and a great surface area. Whereas the edges of graphene oxide sheets crumple during the process of oxidation. Thus, GO sheet has been exfoliated fruitfully from graphite powder containing arranged layers of graphene [46,47]. EDX analysis shows that GO contains about 34.14 atomic % of C and 65.18 atomic % of O Figure 5b.

Figure 5c represents the SEM image of GO-COOH. A comparison with GO to the surface of GO-COOH has asymmetrical wrinkles and a rough surface. It can be observed from GO-COOH image that the layers have been separated, which is possibly due to carboxylic groups (-COOH) generation on its surface during oxidation process. On the other hand, the active sites (-COOH groups) are sufficiently shown in the GO sheets. These active sites can adequately exposed increase the interaction of GO-COOH adsorbent with metal ions such as Cr and Pb [48]. According to EDX analysis, GO-COOH contains about 71.04 atomic % of C and 28.96 atomic % of O Figure 5d.

Figure 5e,f depicted the TEM micrograph of GO and GO-COOH, respectively. TEM images showed fewer structural difference between pure and carboxylated GO. The morphology of GO remained same before and after modification except decrease in aggregation of sheets. This may be attributed to negative charge generated due to ionization of carboxylic acid which causes separation of sheet.

### 3.2. Batch Experiment

#### 3.2.1. Comparison of GO and GO-COOH Potential for Pb and Cr Adsorption

To assess the adsorption potential of both, GO and GO-COOH for Pb and Cr, a preliminary study was conducted using fix dose of both adsorbents (0.05 g) and shaken for 4 h at the shaking speed of 180 rpm. The different initial metal concentrations (0, 5, 25, 50, 100,200 and 300 mg L^−1^) were used for both Pb and Cr and Equation (2) was used to calculate experimental metals adsorption capacities (Supplementary Table S1). The GO-COOH showed high adsorption potential for both Pb and Cr at each initial metal concentration level compared to GO. It can be attributed to two main reasons. Firstly, the surface area of GO after modification has increased significantly. Secondly, the carboxylic acid groups have more metal sorption capacity as compared to hydroxyl groups [29], as our GO-COOH has abundant carboxylic groups. The maximum adsorption capacity was recorded at 100 mg L^−1^ for both Pb and Cr and increased significantly (*p* < 0.05) from 200 mg g^−1^ with GO to 282 mg g^−1^ with GO-COOH in case of Pb. While, in case of Cr enhanced from 194 mg g^−1^ with GO to 276 mg g^−1^ with GO-COOH. As GO-COOH was found extensively efficient compared to GO, so for the next whole batch experiment only GO-COOH was evaluated for both Pb and Cr adsorption at a fix metal concentration of 100 mg L^−1^).

#### 3.2.2. Effect of Different GO-COOH Dose

**Figure 6:**
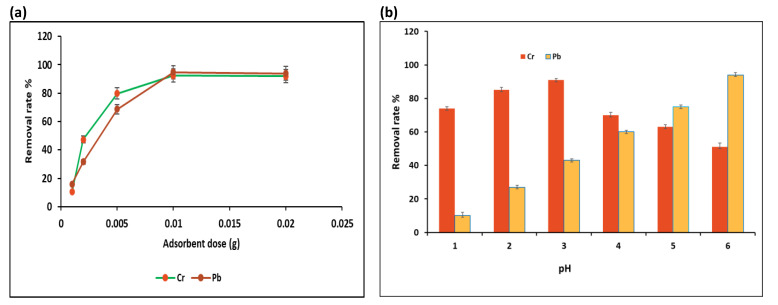
Removal (%) of Pb and Cr via GO-COOH as affected by initial adsorbent dose (**a**) and solution Ph (**b**) (C_0_ = 100 mg/L, Temp. = 25 °C, shaking time = 60 min).

The influence of change in GO-COOH dose on elimination efficiency of Pb (II) and Cr (VI) ions has been represented in Figure 6a. The GO-COOH dose was varied in the 0.001–0.02 g range and 100 mg/L fixed concentration for both metals was adjusted in 25 mL solution volume using contact time of 4 h at 25 °C at the shaking speed of 180 rpm. Based on the results, extracted percentages were observed to be vastly increased when the quantity of adsorbent was increased. Enhancing the quantity and availability of functional moieties on GO-COOH surface is associated with increased metallic removal [47,49]. While the most noteworthy extraction level of Pb (II) (94.7%) and Cr (VI) (92.3%) were achieved at 0.01 g of adsorbent dose. An increase in the amount of adsorbent might result in GO-COOH aggregation, which will make up the bulk of the reactive sites for the adsorbed species and adsorption opaque [50]. Furthermore, as the dose of GO-COOH was further increased the adsorption capacity for both Cr (VI) and Pb (II) does not rise, because adsorption has reached saturation hence adding further adsorbent just makes amount of the adsorption sites waste. Such tendency might also be due to the nanocomposite particles aggregation during the process of adsorption which presents a direct reduction within intact nanocomposite surface area on exposure to metal particles [2,51]. Consequently, 0.01 g dose was selected as optimal adsorbent dosage for Cr (VI) and Pb (II) in terms of removal efficacy and practicability.

#### 3.2.3. Effect of Different pH

An effective adsorbent ought to have the option to bear extreme natural circumstances. The most often concerned boundaries are the ionic strength and pH level of the contaminated media. For the adsorption of negatively charged contaminants, its pH level is crucial because it influenced the deprotonation of-COOH and -OH groups [52]. The pH solution has a substantial role in regulating sorption of intended metal ions, which might be due to chemical structure of metallic ions in the solution or protonation effect by donor atoms within adsorbent [49]. Figure 4b illustrates the effect of different solutions pH on metal ions elimination. Since both complexes and precipitates of metal ions along with OH were found at pH values greater than 6, so pH values were examined in the range of 1.0–6.0 for Pb (II) Cr (VI). The maximum (94%) Pb removal percentage was achieved at pH 6 and (92%) Cr removal percentage at pH 3. According to Figure 6b the efficiency of the GO-COOH in extracting the Pb and Cr ions was significantly increased with increasing solution pH until both metal ions reached their maximum removal percentage at optimal pH. Overall removal of both metal ions was increased by increasing pH from 2.0 to 6.0. The multiple free sites present on GO-COOH surface are responsible for this trend. The Cr (VI) species extraction shows a clear trend with pH and its maximum quantity removed at pH 3 or removal efficiency curve showed the decline tendency with increasing pH. At pH 3, Cr(VI) was seen to be substantially adsorbed showing highest removal efficiency of 92.10%. For pH 2, its absorption rate was just under 85.03%. Overall removal efficiency began to drop at a pH of about 4. Numerous surface functional groups of the nanocomposite help absorb Cr (VI) [53]. In aqueous phase different Cr (VI) species present such as CrO_4_^2−^, HcrO^−^, and Cr_2_O_7_^2−^. At the point when the pH is high to mildly acidic (1–6), HcrO^4^ is an anionic structure [54]. Similarly, to this, at pH levels under 4, the removal of Cr (VI) was worked on because of the electrostatic association between the HcrO^4^ anions and the GO-COOH nanocomposite are highly electrostatically attracted, and have a substantially larger adsorption capacity [34,55]. Reduction in Pb (II) removal efficiency was noticed with decreasing in pH as predicted in Figure 6b. This least Pb (II) ions removal at low pH is credited to presence of hydronium ion (H_3_O^+^) present on adsorbent surfaces competes with positively charged metallic ions on adsorption sites, which results in a lowest efficiency removal of Pb (II) ions at lower pH values [56]. Moreover, at pH greater than 6, the hydroxides and oxides of lead ions precipitate [57]. Increasing solution pH and reducing [H^+^] might lead to a higher concentration of deprotonated surfaces, increasing adhesion along with Pb (II) ion removal while modifying Cr (VI) species extraction. Variable [H^+^] influences on GO-COOH composite active functional groups as well as metal ions. Because of the high [H^+^] COOH^+^ groups become positively charged and protonated, repelling Cr (VI) and Pb (II) ions and making them less adsorbed. Apparently both metals showed very low evacuation pace at lower pH level (below 2) that might be due to the competition between metal ions and H_3_O^+^ for GO-COOH active sites. While on increasing pH up to 6 the increase in removal efficiency relates to diminishing accessible grouping of H_3_O^+^ and lack of competition for the GO-COOH active sites [40].

### 3.3. Sorption Isotherm Models

Equilibrium studies for metal ions removal from the solution were confirmed through different adsorption isotherm models. Langmuir, Temkin and Freundlich isotherms are most popular models for describing equilibrium solid-liquid system. Based on the Langmuir model, metal adsorption occurs as monolayers on a homogeneous surface.

Langmuir isotherm can be written as in Equation (3)

(3) $$\frac{C_{e}}{q_{e}}=\frac{C_{e}}{Q_{max}}+\frac{1}{K_{L}\;Q_{max}}$$

Here *q_e_* (mg g^−1^) indicates adsorbed quantity of metals at equilibrium, *C_e_* (mg L^−1^) indicates concentration of metals in the supernatant at equilibrium, *K_L_* (L mg^−1^) represents adsorption constants of Langmuir model relative to adsorption energy. *Q_max_* (mg g^−1^) symbolize to the maximum adsorption capacity.

For Langmuir isothermal model, the absorption extent can be predicted from separation factor (R_L_) using the following Equation (4):

(4) $$R_{L}=\frac{1}{1+K_{L}\;C_{o}}$$

where, *C_0_* and *K_L_* represent initial metal ions concentration and Langmuir constant, respectively. The values of R_L_ > 1 indicate sorption is opposing, R_L_ = 1: sorption is straight, R_L_ = 0: sorption is irreversible and 0 < R_L_ < 1: sorption is positive and feasible [58].

The Freundlich isotherm equation can be expressed in its linear form using Equation (5)

(5) $$Inq_{e}=Ink_{f}+\frac{1}{n}In\;C_{e}$$

Where, *q_e_* (mg g^−1^) is the adsorbed metal amount at equilibrium, *C_e_* represents the equilibrium amount of metal ions in the fluid, *K_f_* represents the Freundlich constant. *N* is the slope and *K_f_* is the intercept derivative; these are the indicators of adsorption capacity and adsorption intensity, respectively.

Equation (6) shows the Temkin isotherm equation in its linearized version

(6) $$Q_{e}=\frac{RT}{b_{T}\;}\;In\;K_{T}+\frac{RT}{b_{T}}\;In\;c_{e}$$

where, *R* (8.314 J mol^−1^ K^−1^) represents ideal gas constant. *T* represents temperature (K). *K_T_* (L g^−1^) is the constant for Temkin isotherm, *b_T_* (J mol^−1^) is related to Temkin’s heat of adsorption.

**Figure 7:**
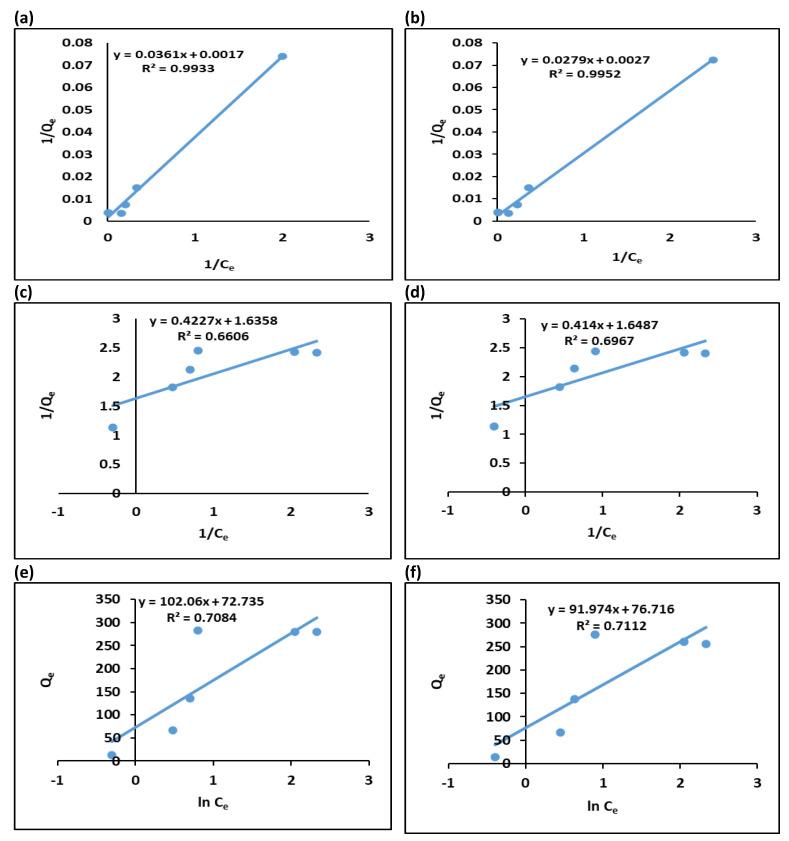
The equilibrium isotherm trends for adsorption of Pb and Cr on to GO-COOH; (**a**) = Langmuir for Pb (II), (**b**) = Langmuir for Cr (VI)), (**c**) = Freundlich for Pb (II) and (**d**) = Freundlich for Cr (VI), (**e**) = Temkin for Pb (II) and (**f**) = Temkin for Cr (VI).

As seen in Figure 7, the Freundlich, Temkin and Langmuir isotherms for adsorption of Pb (II) and Cr (VI) on the GO–COOH at various Pb (II) and Cr (VI) concentrations were plotted. In accordance to the acquired data from these models, the Langmuir model produced the best fit, with the correlation coefficient (*R*^2^) values following the order Langmuir > Temkin > Freundlich isotherm (Table 1). The Langmuir isotherm model exhibited highest correlation coefficients (R^2^) 0.9933 for Pb (II) and 0.9952 for Cr (VI) suited the process of adsorption better as compared to Temkin and Freundlich models. It shows that process of adsorption happened on a homogenous monolayer surface [59]. Yusuf’s study on heavy metal adsorption analyses on graphene-based materials indicated that the Langmuir models better fitted most of the experimental observations [60]. There might be electrostatic interaction between numerous carboxyl groups within GO–COOH and heavy metallic ions, the metal ions adsorption may have increased. GO-COOH gave maximum adsorption capacity (*Q_max_*) of 588.23 and 370.37 mg g^−1^ for Pb (II) and Cr (VI), respectively. Table 1 demonstrates that all the RL values determined for GO-COOH adsorption were below 1, indicating that the process was successful. Additionally, it was noted that the RL values dropped as the initially concentrations increased, indicating that the adsorption became more advantageous as the GO-COOH concentration increase [61]. So, as the RL values of Langmuir model lies between 0 and 1 for both heavy metals, showing that GO-COOH is benefited for adsorption of Pb (II) and Cr (VI) and process of adsorption is feasible [62].

As illustrated in Figure 7c,d the graph of *lnq_e_* vs. *lnC_e_*, The *R^2^* value of Freundlich isotherm was 0.6606 and 0.6967 for Pb (II) and Cr (VI), respectively. Temkin isotherm represented in Figure 7e,f, *q_e_* is plotted vs. *lnCe* shows low *R^2^* = 0.7084 for lead and 0.7112 for chromium ions.

**Figure 8:**
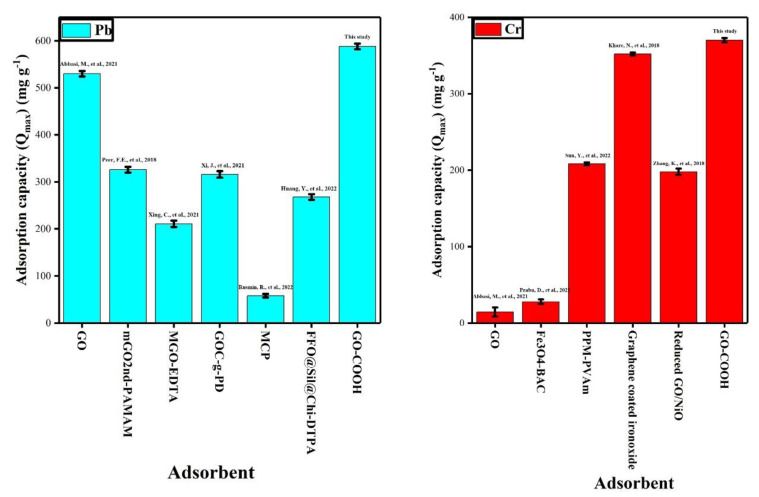
Different adsorbents with their effectiveness for relative metal ions adsorption. (TS represents the results of this study). Abbasi, M., et al., 2021 [63]; Peer, F.E., et al., 2018 [64], Xing, C., et al., 2021 [65], Xi, J., et al., 2021 [66], Rusmin, R., et al., 2022 [67], Huang, Y., et al., 2022 [68], Prabu, D., et al., 2022 [69], Sun, Y., et al., 2022 [70], Khare, N., et al., 2018 [71], Zhang, K., et al., 2018 [72].

The lower regression coefficient (*R^2^*) values for the Freundlich and Timken isotherms indicate that none can match the experimental data. As compared to Cr (VI) the GO-COOH showed better capacity to adsorb Pb (II). The maximum adsorption capacities showed by GO-COOH for both metal ions are greater than those most of the adsorbents previously published in the literature (Figure 8).

The values are presented as the average of 3 replicates.

### 3.4. Sorption Kinetics and Kinetic Models

**Figure 9:**
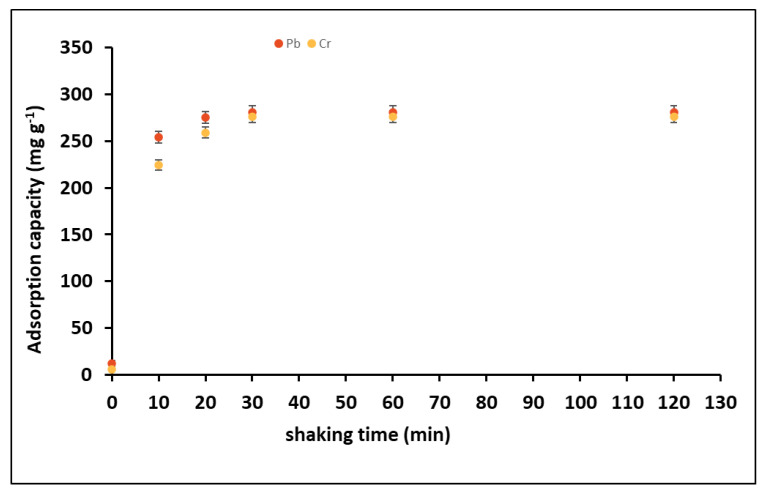
Sorption kinetics of Cr and Pb on GO-COOH.

We changed contact times ranging from 0 to 120 min while other batch conditions (metal concentration 100 mg kg^−1^, pH 6 for Pb and 3 for Cr, adsorbent dose 0.01 g) were kept constant and unraveled the contact time-based change in adsorption capacity of GO-COOH Figure 9. In the first 10 min, the rate of adsorption sharply increased and highest adsorption rates of GO-COOH were achieved in the initial 30 min for both metals. After that, removal efficiency gradually became constant due to achievement of equilibrium. This is due to the abundance of active sites on GO-COOH at first, which promotes metal ions do interact with more and quicker. When more active sites are filled over time, the number of efficient sites for adsorbing heavy metals decreases. However, when metal concentrations in solution reduced by sorption, the transit rates of metal ions to the surfaces of GO-COOH decrease. Consequently, the increase in Pb (II) and Cr (VI) sorption became slower and slower with time. Because when most active sites on GO-COOH are occupied the number of active sites for adsorbing metal ions decreases [31,73].

**Figure 10:**
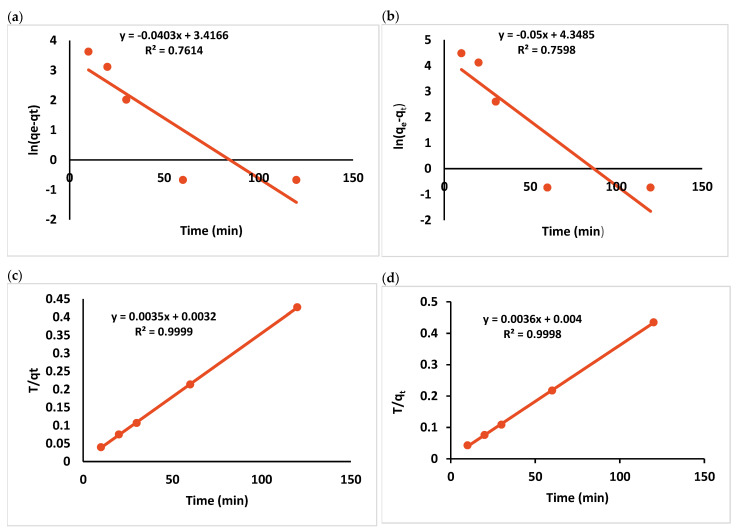
Pseudo-first order for Pb and Cr (**a**,**b**), pseudo-second order for Pb and Cr (**c**,**d**).

Furthermore, observed concentrations of metals ion solutions gradually decline with sorption, lowering heavy metal ion transport rate towards the surfaces of GO-COOH. Hence, an increase in Cr (VI) and Pb (II) adsorbed amounts on GO-COOH became slower and slower with time. So, when majority of the active sorption sites on GO-COOH are occupied, the heavy metals ions adsorption on GO-COOH achieved equilibrium after 30 min. The kinetics data of both metal ions adsorption on GO-COOH was modelled using the pseudo-first order and pseudo-second order (Figure 10a–d). The following equations represent these models

(7) $$q_{t}=q_{e}\left(1-e^{-k_{1}t}\right)$$

(8) $$q_{t}=(k_{2}{q_{e}}^{2}t)/(1+k_{2}q_{e}t)$$

where K_1_ corresponds to the pseudo-first order (min^−1^) and K_2_ corresponds to the pseudo-second-order rate constants and is expressed as (g mg^−1^ min^−1^). The equilibrium adsorption capacity and adsorbed metal amount at time t (min) are expressed as *q_e_* (mg g^−1^) and *q_t_* (mg g^−1^), respectively. The related fitting results and parameters of both models have been presented in Table 2. Our findings demonstrate that the pseudo-second-order model’s *R*^2^ values for both metals on GO-COOH adsorption are all greater than pseudo-first-order model’s which indicate that the first order model could not be properly explain the kinetics of Pb (II) and Cr (VI) on to the modified adsorbent. As shown by a significantly higher coefficient of correlation (*R*^2^ = 0.9999) for Pb (II) and (*R*^2^ = 0.9998) for Cr (VI), the pseudo-second order may adequately characterize the kinetic. Both theoretical and experimental adsorption values were in good accordance, and the R^2^ value is quite close to unity [59]. Secondly, pseudo-second-order model computed *q_e_* values (285.71 and 277.77 mg g^−1^) are closer to experimental values (281.5 and 276.5 mg g^−1^) (Supplementary Table S1) than pseudo-first-order model. Furthermore, calculated values (Qe,cal) derived from linear adsorption capacity are used for first-order rate expression (Qe,exp). However, calculated values derived from linear plots are substantially less than the observed adsorption capacity (Qe,exp) for first-order rate expression [48].

As a result, the pseudo-second-order model could appropriately describe the experimental adsorption data of Pb (II) and Cr (VI) on GO-COOH in our study. Therefore, Pb (II) and Cr (VI) adsorption on GO-COOH is an approach of surface adsorption including chemisorption and the adsorption process is chemically rate controlled. Where removal from a solution owing to physicochemical interaction among the two phases. These findings are supported by [58,74].

The values are presented as the average of 3 replicates.

## 4. Conclusions

The present study tries to address the most prevalent problem of heavy metal contamination of water using graphene-based structures for having very high surface area. Raman spectroscopy and XRD analysis confirmed the successful formation of graphene oxide and its carboxylation with chloroacetic acid. The increase in surface area after modification of graphene was confirmed by BET analysis (Supplementary Table S2). The as-synthesized GO and GO-COOH were applied for remediation of water polluted with Pb (II) and Cr (VI). The highest adsorption capacity obtained for Pb (II) and Cr (VI) was 588.23 and 370.37 mg g^−1^, respectively, with GO-COOH. Langmuir adsorption isotherm and Pseudo second order model were used to fit experimental data for both metals. The excellent adsorption capacity of GO-COOH along with ease of synthesis with low-cost chemicals make it excellent candidate for industrial scale application for effective Pb (II) and Cr (VI) removal from wastewater. To scale up the application of the present technique, we are exploring novel methods to synthesize graphene and its structures. Implementation of this technique may help reduce heavy metals inflicted diseases. Further research is needed on the facile synthesis of GO and its modified structures to make it more economical for industrial applications on which we are working.

## Figures and Tables

**Table 1:** Adsorption isotherm models parameters.

Metals	Freundlich			Langmuir				Temkin		
	***n***	***K_f_*** **(mg g^−1^)**	***R*** **^2^**	***Q_m_*** **(mg g^−1^)**	***b_L_***	***R_L_*** **(Lmg^−1^)**	***R*** **^2^**	***b_T_*** **(J mol^−1^)**	***K_T_*** **(L g^−1^)**	***R*** **^2^**
Pb	2.36	5.13	0.66	588.23	0.04	0.33	0.99	24.28	2.03	0.70
Cr	2.41	5.2	0.69	370.37	0.09	0.5	0.99	26.95	2.71	0.71

**Table 2:** Adsorption kinetics parameters for metals adsorption on GO-COOH.

Adsorbent	Heavy Metal	Pseudo-First Order			Pseudo-Second Order		
		*q_e_*	*K*_1_	*R* ^2^	*q* ^2^ *_e_*	*K_2_*	*R* ^2^
		(mg g^−1^)	(min^−1^)		(mg g^−1^)	(min^−1^)	
GO-COOH	Pb	30.465	0.00034	0.7614	285.71	0.0038	0.999
	Cr	77.362	0.00042	0.7598	277.77	0.0032	0.9998

## Data Availability

Not applicable.

## Supplementary Materials

The following supporting information can be downloaded at: https://www.mdpi.com/article/10.3390/ijerph191710610/s1; Table S1: Comparison of GO and GO-COOH potential for adsorption capacity (Qe) of Pb and Cr from the contaminated water; Table S2: The Brunauer-Emmett-Teller (BET) surface areas, pore volume and average pore size of GO and GO-COOH.
