# Supplementary material for: Synthesis of Functionalized Carboxylated Graphene Oxide for the Remediation of Pb and Cr Contaminated Water
Source: Int J Environ Res Public Health. 2022 Aug 25;19(17):10610. doi: 10.3390/ijerph191710610 (PMC9518387; doi:10.3390/ijerph191710610)
Supplement: Supplementary file 1 [file ijerph-19-10610-s001.zip › ijerph-1803788-supplementary.pdf]

Supplementary Table S1

Comparison of GO and GO-COOH potential for adsorption capacity (Q_e) of Pb and Cr from the contaminated water

Adsorbent	Initial concentration (mg L^{-1})	(Q_e) Pb (mg g^{-1})	(Q_e) Cr (mg g^{-1})
GO	5	12.1i	10.33 i
	25	51.92 g	48.11 g
	50	122.23 e	119.24 e
	100	200.21 c	194.13 c
	200	196.66 c	192.32 c
	300	196.33 c	189 c
GO-COOH	5	23.83 h	20.93 h
	25	76.65 f	71.36 f
	50	155.76 d	152.36 d
	100	282.16 a	276.12 a
	200	274.33 ab	273.21 ab
	300	271.66 cb	270.01 b

The values are the mean of three replicates. The statistical letters in the same column represents the significant difference between the values.

Supplementary Table S2

The Brunauer-Emmett-Teller (BET) surface areas, pore volume and average pore size of GO and GO-COOH

	BET Surface area (m^2/g)	Total pore volume (dm^3/g)	Average pore size (nm)
GO	29.88	(0.00015)	16.41
GO-COOH	52.79	(0.00013)	20.38
